# Association of systemic immune-inflammation index with all-cause and cardio-cerebrovascular mortality in individuals with diabetic kidney disease: evidence from NHANES 1999-2018

**DOI:** 10.3389/fendo.2024.1399832

**Published:** 2024-11-26

**Authors:** Manhuai Zhang, Siyang Ye, Jianbo Li, Meng Zhang, Li Tan, Yiqin Wang, Peichen Xie, Huajing Peng, Suchun Li, Sixiu Chen, Qiong Wen, Kam Wa Chan, Sydney C. W. Tang, Bin Li, Wei Chen

**Affiliations:** ^1^ Department of Nephrology, The First Affiliated Hospital, Sun Yat-sen University, Guangzhou, China; ^2^ National Health Commission (NHC) Key Laboratory of Clinical Nephrology (Sun Yat-sen University) and Guangdong Provincial Key Laboratory of Nephrology, Guangzhou, China; ^3^ School of Chinese Medicine, Hong Kong Baptist University, Hong Kong, Hong Kong SAR, China; ^4^ Division of Nephrology, Department of Medicine, LKS Faculty of Medicine, The University of Hong Kong, Hong Kong, Hong Kong SAR, China

**Keywords:** systemic immune-inflammation index, diabetes mellitus, diabetic kidney disease, population-based study, NHANES, all-cause mortality, cardio-cerebrovascular disease mortality

## Abstract

**Background:**

Emerging evidence suggests a potential role of immune response and inflammation in the pathogenesis of diabetic kidney disease (DKD). The systemic immune-inflammation index (SII) offers a comprehensive measure of inflammation; however, its relationship with the prognosis of DKD patients remains unclear.

**Methods:**

Using data from the National Health and Nutrition Examination Survey (NHANES) spanning 1999 to 2018, this cross-sectional study involved adults diagnosed with DKD. Cox proportional hazards models were utilized to assess the associations between SII and all-cause or cardio-cerebrovascular disease mortality. Additionally, restricted cubic spline, piecewise linear regression, and subgroup analyses were performed.

**Results:**

Over a median follow-up duration of 6.16 years, 1338 all-cause deaths were recorded. After adjusting for covariates, elevated SII levels were significantly associated with increased risks of all-cause and cardio-cerebrovascular disease mortality. Specifically, per one-unit increment in natural log-transformed SII (lnSII), there was a 29% increased risk of all-cause mortality (*P* < 0.001) and a 23% increased risk of cardio-cerebrovascular disease mortality (*P* = 0.01) in the fully adjusted model. Similar results were observed when SII was analyzed as a categorical variable (quartiles). Moreover, nonlinear association was identified between SII and all-cause mortality (*P* < 0.001) through restricted cubic spline analysis, with threshold value of 5.82 for lnSII. The robustness of these findings was confirmed in subgroup analyses. Likewise, the statistically significant correlation between SII levels and cardio-cerebrovascular disease mortality persisted in individuals with DKD.

**Conclusion:**

Increased SII levels, whether examined as continuous variables or categorized, demonstrate a significant association with elevated risks of all-cause and cardio-cerebrovascular disease mortality among DKD patients. These findings imply that maintaining SII within an optimal range could be crucial in reducing mortality risk.

## 1 Introduction

In recent decades, the surge in diabetes mellitus (DM) cases has propelled it into a critical global health concern, imposing substantial economic burdens worldwide (1). Among its complications, diabetic kidney disease (DKD) looms large, affecting individuals with both type 1 and type 2 DM. Patients with DKD, especially those receiving dialysis, endure a substantial symptom burden and frequent hospital admissions stemming from prevalent comorbidities such as hypertension, coronary artery disease, congestive heart failure, and cerebrovascular disease (2–6). These comorbidities frequently contribute to psychological issues, disabilities, and substantial healthcare costs, markedly impairing patients’ quality of life. Despite current clinical management strategies, which include renin-angiotensin system blockade and meticulous control of hypertension, hyperglycemia, and dyslipidemia, DKD remains a primary contributor to end-stage renal disease (ESRD) necessitating renal replacement therapy (1). The ongoing therapeutic hurdles highlight the pressing necessity for a more profound understanding of DKD’s pathophysiological intricacies, from its onset to advanced renal failure, to identify potential risk factors for screening and intervention. Bridging this knowledge gap is essential for developing novel and effective strategies to prevent and manage the progression of DKD in clinical practice.

Recent investigations have implicated various factors, including metabolic disruptions and hemodynamic irregularities triggered by hyperglycemia and insulin resistance (IR), in the pathogenesis of DKD (7–9). Moreover, IR is closely associated with chronic low-grade inflammation marked by heightened levels of mediators like interleukin-1, interleukin-6, and tumor necrosis factor-α (10, 11). The evolving understanding of DKD portrays it as a disorder driven by metabolic and immunological interplay. Both systemic and localized renal inflammation are recognized as pivotal in DKD progression (12), wherein numerous novel pro-inflammatory signaling pathways have been implicated, including the NOD-like receptor family pyrin domain-containing 3 (NLRP3) inflammasome activation (13), the nuclear factor kappa B (NF-κB) signaling pathway (14), toll-like receptor 4 (TLR4) signaling pathway (15), adenosine 5′-monophosphate-activated protein kinase signaling pathway (16), and the hypoxia-inducible factor-1 signaling pathway (17).

The diabetic microenvironment, marked by hyperglycemia, fluctuating glucose levels, and IR, triggers both systemic and localized inflammatory responses through the TLR4/NF-κB/NLRP3 pathway. These cascades activate platelets, an atypical first-line inflammatory biomarker that may attach to leukocytes and endothelial cells, modifying their pro-inflammatory activities (18–20). For instance, platelet activating factor (PAF), a pro-inflammatory mediator significantly increased by activated platelets, exaggerates leukocyte chemotaxis, complement activation, reactive oxygen species and eicosanoids production in DKD (21–23). PAF also stimulates lymphocytes to produce immunoglobulins and elevates circulating pro-inflammatory cytokines such as interleukin-1, interleukin-6, and tumor necrosis factor-α (24). These cytokines not only amplify platelet activation but also co-stimulate lymphocytes, further exacerbating renal inflammation (25–27). Platelet-derived platelet factor 4 (PF4), a potent chemoattractant produced by platelet that promotes neutrophil adhesion to endothelial cells and lymphocyte chemotaxis (21, 28), has been found to be markedly elevated in DKD patients with macroalbuminuria (23). Neutrophils, which comprise the majority of white blood cells and are critical in initiating and regulating inflammatory processes, release neutrophil elastase-a key player in chronic inflammation and potential contributor to renal damage in DKD (29). Lymphocytes are inflammatory mediators that do have regulatory or protective functions for preventing the progression of chronic kidney disease (30). More importantly, DKD patients exhibit significantly higher neutrophils and platelet counts alongside notably lower lymphocyte counts compared to healthy populations (29–33), indicating heightened inflammation and an imbalance in immune regulation. Taken together, the diabetic milieu orchestrates a broad variety of inflammatory responses, including secretion of pro-inflammatory cytokines, platelet-lymphocyte interaction, and platelet-neutrophil interaction, all of which synergistically contribute to the deterioration of renal function. These findings collectively underscore the critical role of inflammation and immune cells interaction in driving DKD progression.

The systemic immune-inflammation index (SII), an innovative inflammatory marker derived from platelet count × neutrophil count/lymphocyte count, has emerged as a comprehensive measure of inflammation. Initially utilized to assess prognosis in hepatocellular carcinoma patients (34), SII has shown prognostic utility in various cancers and is recognized for its precision in gauging inflammatory status. Recent studies have further linked higher SII with increased incidence of metabolic syndrome (35), cardiovascular disease (36), nonalcoholic fatty liver disease (37), DM (38), urinary albumin excretion (39), diabetic retinopathy (40), and other DM-related complications (41, 42). Moreover, prospective cohort studies have associated SII with elevated risks of cardiovascular, cardio-cerebrovascular, and all-cause mortality in DM individuals (43) and the general population (44). However, the relationship between SII and mortality outcomes in individuals with DKD remains unexplored. Examining the relationship between SII and long-term mortality risk in individuals with DKD is crucial as it provides a deeper understanding of the impact of inflammation and immune status on the health outcomes of DKD patients. Furthermore, such research provides valuable insights for improving clinical management and intervention approaches for DKD patients. To bridge this gap, the present study investigates the association between SII, a novel index reflecting systemic inflammatory state, and all-cause mortality and cardio-cerebrovascular disease mortality in individuals with DKD, using data from the National Health and Nutrition Examination Survey (NHANES) database.

## 2 Materials and methods

### 2.1 Study population

**Figure 1:**
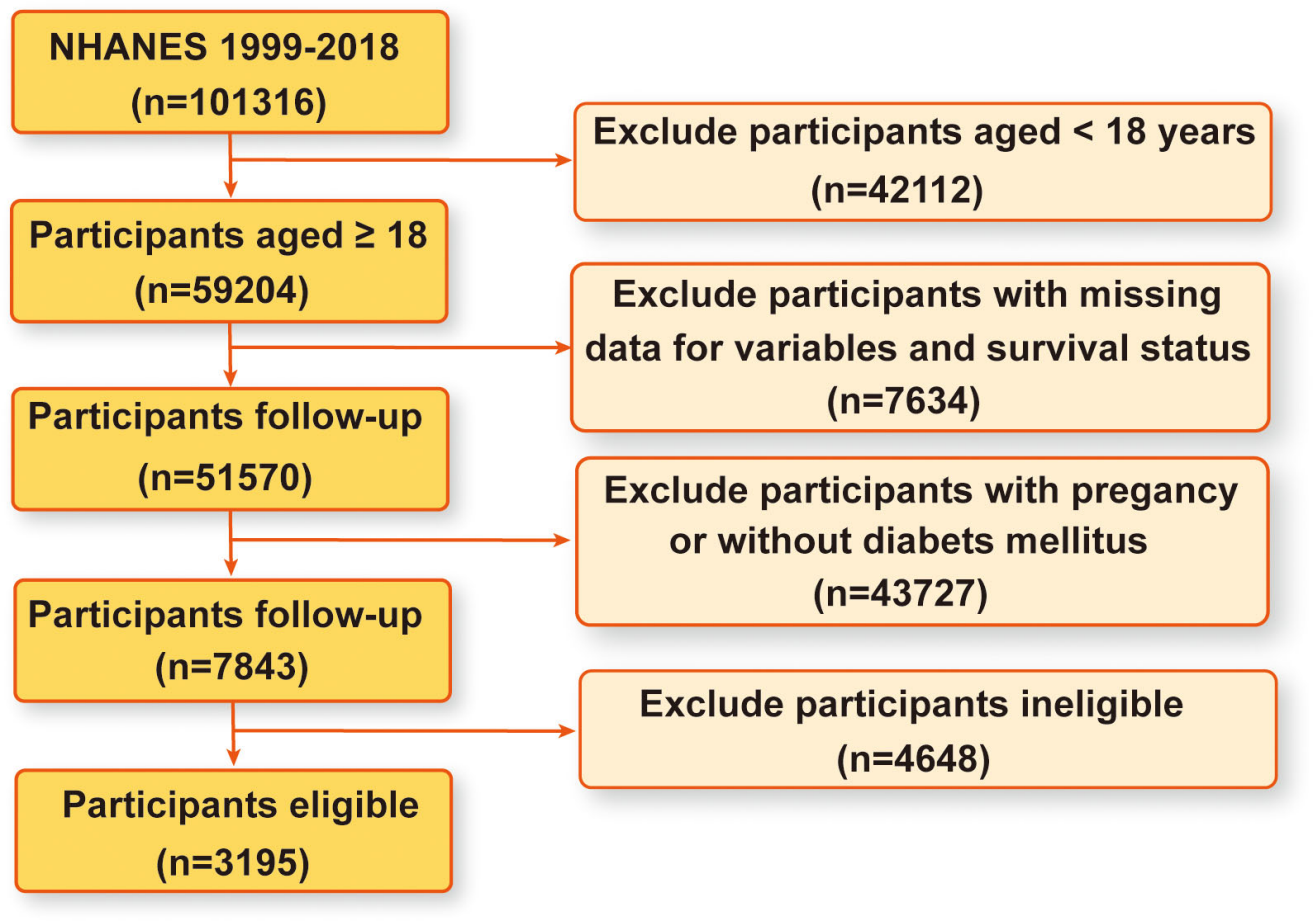
A flow chart of sample selection of eligible participants from the NHANES 1999-2018.

The NHANES database serves as a comprehensive, population-based cross-sectional survey meticulously designed to capture insights into the health and nutritional status of the United States household population (45). Data collection occurs through structured interviews conducted in participants’ homes, complemented by physical examinations conducted at mobile centers and laboratory assessments, all structured within a multistage probability sampling framework. The NHANES protocol has received ethical approval from the National Center for Health Statistics ethics review board, with all participants providing written informed consent. The dataset spans NHANES surveys conducted from 1999 to 2018, encompassing a total of 101316 participants. Through rigorous inclusion criteria, we excluded 42112 individuals under the age of 18, 7634 participants with missing data on pertinent variables and survival status, 43727 individuals who were pregnant or without DM, and 4648 ineligible participants. Consequently, our study enrolled 3195 eligible participants for analysis. **Figure 1** provides a visual representation of the detailed participant selection process.

### 2.2 Definition of systemic immune-inflammation index

Lymphocyte, neutrophil, and platelet counts were determined using automated hematology analysis devices. The SII was derived by multiplying the platelet count by the neutrophil count and then dividing by the lymphocyte count, following established methodologies outlined in prior studies (43).

### 2.3 Definition of diabetes mellitus and diabetic kidney disease

DM was defined as meeting any of the following criteria: (1) a documented diagnosis by healthcare professionals; (2) fasting plasma glucose levels ≥ 7.0 mmol/L; (3) glycosylated hemoglobin levels ≥ 6.5%; or (4) currently taking medications for diabetes management (46, 47). The urine albumin-to-creatinine ratio (UACR) was utilized to determine UACR values. Estimated glomerular filtration rate (eGFR) scores were calculated using the Chronic Kidney Disease Epidemiology Collaboration algorithm. The diagnosis of DKD in patients with diabetes was established based on UACR levels ≥ 30 mg/g and/or eGFR < 60 mL/min/1.73m^2^ (48).

### 2.4 Determination of mortality outcomes

Mortality outcomes were identified using death certificate records obtained from the National Death Index. All-cause mortality was assessed by examining publicly accessible death data linked to the NHANES datasets until December 31, 2019. Cardio-cerebrovascular disease mortality was determined based on the International Classification of Diseases, 10th Revision (ICD-10) codes I00-I09, I11, I13, I20-I51, or I60-I69.

### 2.5 Definition of other variables

This investigation encompassed various covariates potentially influencing the relationship between SII and DKD. Demographic parameters comprised age, sex, race, body mass index (BMI), poverty income ratio, education, smoking status, alcohol, physical activity, and frailty. Ethnicity categories were delineated as White, Black, Hispanic, Mexican, and others. BMI (kg/m^2^) was calculated by dividing weight by height squared and categorized according to World Health Organization standards: < 18.5 (underweight), 18.5-24.9 (healthy weight), 25-29.9 (overweight), and ≥ 30 (obese) (49). Poverty income ratio is a pre-defined continuous variable in NHANES and is based on the ratio of the family household income to the poverty level set by the US Department of Health and Human Services. Educational level was stratified into less than high school, high school or equivalent, and college or above. Smoking habits were classified as never, former, or current. Alcohol consumption patterns were categorized as never, former, mild, moderate, or heavy drinking. Physical activity was categorized as vigorous, moderate or no. Frailty status was constructed based on the previous standard procedure (50). The frailty index consisted of 49 deficits with a value ranging from 0 (no frailty) to 1 (frailty) according to the severity of the deficit, and a cut-off point of 0.21 on the frailty index value divided participants into two groups of frailty or not. Health risk factors included hypertension, hyperlipidemia, cardiovascular disease (CVD), and SII status. DM-related treatments encompassed the usage of anti-inflammatory and anti-diabetic medications. Previous disease history, including hypertension, hyperlipidemia, and CVD, was obtained from health-related questionnaires or test results. Hypertension was defined as meeting at least one of the following criteria: (1) systolic blood pressure ≥ 140 mmHg and/or diastolic blood pressure ≥ 90 mmHg after repeated examination or a prior diagnosis by a physician (51); (2) self-reported history of hypertension; (3) current use of antihypertensive medications. Hyperlipidemia was defined by total cholesterol ≥ 240 mg/dL, triglycerides ≥ 200 mg/dL, LDL-cholesterol ≥ 160 mg/dL, HDL-cholesterol < 40 mg/dL, or a physician’s diagnosis. Due to the variability in participants’ medication regimens, anti-inflammatory or anti-diabetic therapy was dichotomized into “no” (participants not taking anti-inflammatory or anti-diabetic drugs) and “yes” (participants receiving anti-inflammatory or ani-diabetic drugs). Detailed measurement techniques for these variables are accessible at www.cdc.gov/nchs/nhanes/.

### 2.6 Statistical analyses

Normally distributed continuous variables were reported as weighted means ± standard error, while non-normally distributed continuous variables were reported as median [Interquartile range (IQR)]. Categorical variables were expressed as frequency and percentage. We utilized weighted Student’s t-test for normally distributed continuous variables, Mann-Whitney U test for non-normally distributed continuous variables, and chi-square test for categorical variables to compare baseline characteristics between survivors and deceased DKD subjects.

Associations between SII and the risk of all-cause mortality or cardio-cerebrovascular disease mortality were assessed using multivariate Cox proportional hazard models to estimate hazard ratios (HRs) and 95% confidence intervals (CIs). We examined the proportional hazard assumption using Schoenfeld residual methods. Given the right-skewed distribution of SII, the variable was assessed in its continuous form after applying a natural log-transformation (lnSII), and the lnSII variable was grouped into four quartiles, which were included in models as both continuous and categorical variables. The first quartile of lnSII (Q1) served as the reference group, with median values assigned to each category to evaluate linear trends. Baseline variables clinically relevant to prognosis were adjusted in the multivariable Cox proportional hazard model. Model 1 adjusted for sex, age, and race, while model 2 further adjusted for BMI, poverty income ratio, education level, smoking status, alcohol consumption, physical activity, frailty status, hypertension, hyperlipidemia, cardiovascular disease, usage of anti-inflammatory drugs and anti-diabetic drugs. To explore dose-response associations between lnSII and mortality, restricted cubic spline (RCS) with 4 knots (5th, 35th, 65th, 95th) was employed. If nonlinear associations are detected, the “segmented” package was applied to identify inflection points and perform segmented Cox proportional hazard regression.

Stratified analyses were conducted by age (< 60 years and ≥ 60 years), sex (male and female), BMI (≥ 25 and < 25), hypertension (yes and no), hyperlipidemia (yes and no), usage of anti-inflammatory drugs (yes and no), usage of anti-diabetic drugs (yes and no), and frailty status (yes and no). Potential interactions were tested by likelihood ratio tests. Statistical analysis was performed with R software, Version 4.2.1. Two-sided *P* < 0.05 was considered statistically significant.

## 3 Results

### 3.1 Baseline characteristics of study participants

A total of 3195 participants diagnosed with DKD were included in the study, with a median age of 67.00 years and a gender distribution of 1720 (53.8%) male patients and 1475 (46.2%) female patients. Among these participants, 1857 (58.1%) were classified as survivors during a median follow-up period of 6.16 years. **Table 1** presents the demographic and clinical characteristics of the participants stratified by all-cause mortality. Significant differences were observed between survivors and deceased subjects in terms of age, sex, race, BMI, poverty income ratio, education level, smoking status, alcohol consumption, physical activity, frailty status, hypertension, hyperlipidemia, cardiovascular disease, and SII (all *P* < 0.05). However, there were no significant differences in the use of anti-inflammatory drugs and anti-diabetic drugs between survivors and deceased DKD subjects.

**Table 1:** Baseline demographic and clinical characteristics of study populations by presence of DKD.

Variables	All participants	Survivors during follow-up	Deceased during follow-up	*P* value
Number of participants	3195	1857	1338	
Age (median [IQR])	67.00 [59.00, 76.50]	63.00 [53.00, 72.00]	74.00 [65.00, 80.00]	<0.001
Sex (%)				0.009
Female	1475 (46.2)	894 (48.1)	581 (43.4)	
Male	1720 (53.8)	963 (51.9)	757 (56.6)	
Race (%)				<0.001
White	1234 (38.6)	570 (30.7)	664 (49.6)	
Black	788 (24.7)	475 (25.6)	313 (23.4)	
Hispanic	260 (8.1)	191 (10.3)	69 (5.2)	
Mexican	642 (20.1)	412 (22.2)	230 (17.2)	
Others	271 (8.5)	209 (11.3)	62 (4.6)	
Body mass index (%)				<0.001
Obese (≥ 30)	1837 (57.5)	1153 (62.1)	684 (51.1)	
Overweight (≥ 25 to < 30)	881 (27.6)	482 (26.0)	399 (29.8)	
Healthy weight (≥ 18.5 to < 25)	465 (14.6)	219 (11.8)	246 (18.4)	
Underweight (< 18.5)	12 (0.4)	3 (0.2)	9 (0.7)	
Poverty income ratio (%)				<0.001
≥ 3.0	709 (22.2)	463 (24.9)	246 (18.4)	
1.0-3.0	1749 (54.7)	962 (51.8)	787 (58.8)	
≤ 1.0	737 (23.1)	432 (23.3)	305 (22.8)	
Education (%)				<0.001
College or above	1157 (36.2)	751 (40.4)	406 (30.3)	
High school or equivalent	708 (22.2)	421 (22.7)	287 (21.4)	
Less than high school	1330 (41.6)	685 (36.9)	645 (48.2)	
Smoking status (%)				<0.001
Current	482 (15.1)	279 (15.0)	203 (15.2)	
Former	1199 (37.5)	624 (33.6)	575 (43.0)	
Never	1514 (47.4)	954 (51.4)	560 (41.9)	
Alcohol (%)				<0.001
Heavy	308 (9.6)	223 (12.0)	85 (6.4)	
Moderate	215 (6.7)	155 (8.3)	60 (4.5)	
Mild	813 (25.4)	512 (27.6)	301 (22.5)	
Former	1283 (40.2)	645 (34.7)	638 (47.7)	
Never	576 (18.0)	322 (17.3)	254 (19.0)	
Physical activity (%)				<0.001
No	2190 (68.5)	1200 (64.6)	990 (74.0)	
Moderate	766 (24.0)	478 (25.7)	288 (21.5)	
Vigorous	239 (7.5)	179 (9.6)	60 (4.5)	
Frailty (%)				<0.001
Yes	1974 (61.8)	1008 (54.3)	966 (72.2)	
No	1221 (38.2)	849 (45.7)	372 (27.8)	
Hypertension (%)				0.001
Yes	2594 (81.2)	1472 (79.3)	1122 (83.9)	
No	600 (18.8)	385 (20.7)	215 (16.1)	
Hyperlipidemia (%)				0.62
Yes	2853 (89.3)	1663 (89.6)	1190 (88.9)	
No	342 (10.7)	194 (10.4)	148 (11.1)	
Cardiovascular disease (%)				<0.001
Yes	1117 (35.0)	484 (26.1)	633 (47.3)	
No	2078 (65.0)	1373 (73.9)	705 (52.7)	
Taking anti-inflammatory drugs (%)				0.496
Yes	261 (8.2)	146 (7.9)	115 (8.6)	
No	2934 (91.8)	1711 (92.1)	1223 (91.4)	
Taking anti-diabetic drugs (%)				0.429
yes	2246 (70.3)	1316 (70.9)	930 (69.5)	
no	949 (29.7)	541 (29.1)	408 (30.5)	
Systemic immune-inflammation index (%)				<0.001
Q1 [17.38, 362.62)	799 (25.0)	506 (27.2)	293 (21.9)	
Q2 [362.62, 524.36)	799 (25.0)	492 (26.5)	307 (22.9)	
Q3 [524.36, 753.82)	798 (25.0)	463 (24.9)	335 (25.0)	
Q4 [753.82, 11700.00)	799 (25.0)	396 (21.3)	403 (30.1)	

Data presented as numbers (percentages) unless otherwise indicated. All estimates accounted for complex survey designs, and all percentages were weighted. IQR, interquartile range.

### 3.2 Associations between SII and mortality

As depicted in **Table 2**, the natural logarithm of the SII exhibited a significant association with an elevated risk of all-cause mortality in the crude model (HR = 1.31, 95% CI = 1.19-1.45). Following multivariable adjustment, this association remained robust and statistically significant in both Model 1 (HR = 1.36, 95% CI = 1.23-1.50) and Model 2 (HR = 1.29, 95% CI = 1.17-1.42). Moreover, we transformed lnSII from a continuous variable to a categorical variable and constructed several models to assess the independent effects of SII on mortality. Compared to individuals in the first quartile of lnSII (Q1), those in the fourth quartile (Q4) exhibited notably higher multivariate-adjusted HRs, as evidenced by Model 1 (HR = 1.66, 95% CI = 1.43-1.94, *P* for trend < 0.001) and Model 2 (HR = 1.58, 95% CI = 1.35-1.84, *P* for trend < 0.001). Similarly, this statistically significant association with lnSII in DKD individuals persisted for cardio-cerebrovascular disease mortality (**Table 2**). Each one-standard deviation increase in lnSII was associated with a 17% elevated risk of cardio-cerebrovascular disease mortality in the crude model (HR = 1.24, 95% CI = 1.05-1.46). After adjusting for multiple variables, this correlation remained strong and statistically significant in both Model 1 (HR = 1.29, 95% CI = 1.10-1.53) and Model 2 (HR = 1.23, 95% CI = 1.04-1.45). In contrast to individuals in the first quartile of lnSII (Q1), those in the fourth quartile (Q4) showed significantly higher multivariate-adjusted HRs, as demonstrated by Model 1 (HR = 1.40, 95% CI = 1.09-1.81, *P* for trend = 0.003) and Model 2 (HR = 1.32, 95% CI = 1.02-1.71, *P* for trend = 0.021).

**Table 2:** Multivariable Cox proportional hazard model analyses for all-cause mortality and cardio-cerebrovascular disease mortality among DKD participants.

Levels of lnSII	Number of deaths/Total participants	Crude model		Model 1		Model 2	
		HR (95% CI)	P value	HR (95% CI)	P value	HR (95% CI)	P value
All-cause mortality							
Continuous	1338/3195	1.31 (1.19, 1.45)	<0.001	1.36 (1.23, 1.50)	<0.001	1.29 (1.17, 1.42)	<0.001
Q1	293/799	Ref		Ref		Ref	
Q2	307/799	0.98 (0.84, 1.15)	0.827	1.09 (0.93, 1.28)	0.310	1.11 (0.94, 1.31)	0.213
Q3	335/798	1.12 (0.96, 1.31)	0.145	1.10 (0.94, 1.29)	0.231	1.08 (0.92, 1.27)	0.331
Q4	403/799	1.49 (1.28, 1.73)	<0.001	1.66 (1.43, 1.94)	<0.001	1.58 (1.35, 1.84)	<0.001
P for trend		<0.001		<0.001		<0.001	
Cardio-cerebrovascular mortality							
Continuous	485/3195	1.24 (1.05, 1.46)	0.01	1.29 (1.10,1.53)	0.002	1.23 (1.04, 1.45)	0.014
Q1	117/799	Ref		Ref		Ref	
Q2	111/799	0.89 (0.69, 1.16)	0.384	0.99 (0.76, 1.29)	0.937	1.02 (0.78, 1.33)	0.887
Q3	122/798	1.03 (0.80, 1.32)	0.839	1.00 (0.78, 1.30)	0.971	0.98 (0.75, 1.27)	0.856
Q4	135/799	1.25 (0.98, 1.60)	0.076	1.40 (1.09, 1.81)	0.009	1.32 (1.02, 1.71)	0.032
P for trend		0.02		0.003		0.021	

SII was assessed in its continuous form after applying a natural log-transformation (lnSII). Crude Model: unadjusted. Model 1: adjust for age, sex, race. Model 2: adjust for age, sex, race, body mass index, poverty income ratio, education level, smoking status, alcohol consumption, physical activity, frailty status, hypertension, hyperlipidemia, cardiovascular disease, usage of anti-inflammatory drugs and anti-diabetic drugs. SII, systemic immune inflammation index; HR, hazard ratio; 95% CI, 95% confidence interval.

### 3.3 Dose-response relationship between SII and mortality

**Figure 2:**
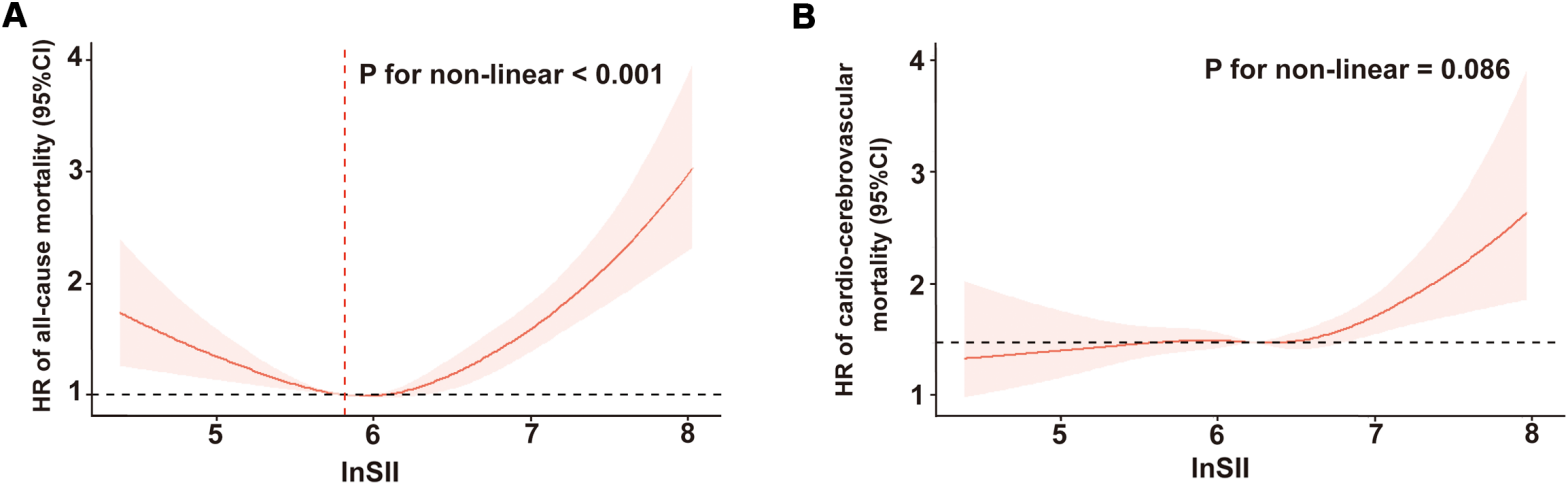
Restricted cubic spline regression of the relationship between lnSII and all-cause mortality **(A)** or cardio-cerebrovascular disease mortality **(B)**. SII was assessed in its continuous form after applying a natural log-transformation (lnSII). SII, systemic immune-inflammation index; HR, hazard ratio; 95% CI, 95% confidence interval.

As illustrated in **Figure 2A**, after adjustment for multiple potential confounders, we observed a statistically significant nonlinear and U-shaped association between the lnSII and all-cause mortality (*P* for nonlinear < 0.001). By contrast, a linear association emerged between lnSII and cardio-cerebrovascular disease mortality (*P* for nonlinear = 0.086, **Figure 2B**). The Segmented Cox proportional hazard model analysis presented in **Table 3** unveiled that the risk of all-cause mortality initially declined (HR = 0.58, 95% CI = 0.43-0.78), reaching its inflection at a lnSII value of 5.82, before subsequently escalating with increasing lnSII levels (HR = 1.69, 95% CI = 1.48-1.93).

**Table 3:** Segmented Cox proportional hazard regression analyses for the effect of SII on all-cause mortality among DKD participants.

	HR (95% CI)	*P* value
lnSII below 5.82		
Crude model	0.51 (0.39, 0.66)	< 0.001
Model 1	0.59 (0.45, 0.77)	< 0.001
Model 2	0.58 (0.43, 0.78)	< 0.001
lnSII above 5.82		
Crude model	1.78 (1.56, 2.04)	< 0.001
Model 1	1.84 (1.61, 2.10)	< 0.001
Model 2	1.69 (1.48, 1.93)	< 0.001

SII was assessed in its continuous form after applying a natural log-transformation (lnSII). Crude Model: unadjusted. Model 1: adjust for age, sex, race. Model 2: adjust for age, sex, race, body mass index, poverty income ratio, education level, smoking status, alcohol consumption, physical activity, frailty status, hypertension, hyperlipidemia, cardiovascular disease, usage of anti-inflammatory drugs and anti-diabetic drugs. SII, systemic immune inflammation index; HR, hazard ratio; 95% CI, 95% confidence interval.

### 3.4 Subgroup analysis

**Figure 3:**
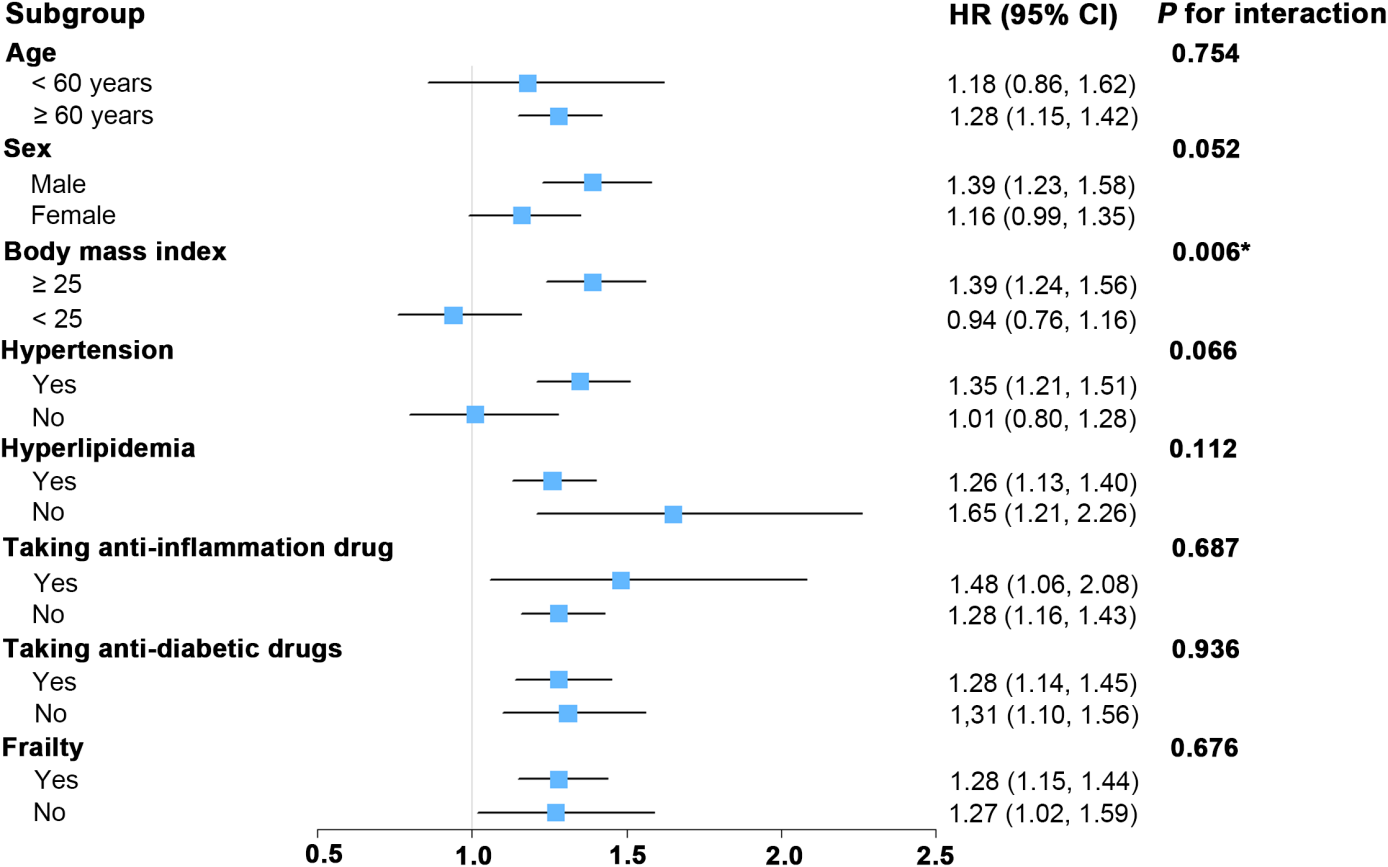
Forest plot for subgroup analysis of associations between lnSII and all-cause mortality. Hazard ratios (HR) were calculated using multivariate Cox proportional hazards models adjusted for variables in model 2 except for the variable used for stratification. SII was assessed in its continuous form after applying a natural log-transformation (lnSII). SII, systemic immune-inflammation index; 95% CI, 95% confidence interval.

As depicted in **Figure 3**, subgroup analyses were conducted to assess whether various demographic and clinical characteristics could influence the relationship between SII and all-cause mortality. Our subgroup analysis revealed significant interaction between SII and BMI (*P* for interaction < 0.05). For those DKD patients with higher BMI (≥ 25), there was a significant association between SII and all-cause mortality. For DKD patients with lower BMI (< 25), the association between SII and risk of death was not significant. Furthermore, the stratified analyses revealed no significant interactions between SII and the stratified components including age, sex, hypertension, hyperlipidemia, taking anti-inflammation drug, taking anti-diabetic drug, and frailty (all *P* for interaction > 0.05). This finding aligns with previously published studies in other population types (52, 53), indicating that these stratified variables did not significantly influence the positive association between SII and all-cause mortality.

## 4 Discussion

In this study, we for the first time conducted a novel investigation into the relationship between the SII and both all-cause mortality and cardio-cerebrovascular disease mortality in individuals diagnosed with DKD using data from the NHANES database. Our analysis unveiled notable findings: a U-shaped correlation between SII levels and the risk of all-cause mortality in DKD individuals was observed, and the inflection point of lnSII with the lowest HR was 5.82, indicating that both excessively low and high concentrations were associated with an increased risk. This relationship remained consistent across various stratified analyses. Additionally, we found a linear correlation between SII levels and the risk of cardio-cerebrovascular disease mortality. Taken together, these findings suggest that SII could serve as a predictive marker for mortality risk in DKD patients and may represent a potential target for interventions aimed at improving health outcomes.

Emerging evidence suggests that chronic immune cell overactivation and subsequent low-grade inflammation may underlie the pathogenesis of DKD and its dire prognosis (15, 54–56). A comprehensive analysis of multiple genome-wide association studies revealed that a significant portion of single nucleotide polymorphisms associated with DKD are directly or indirectly linked to inflammation and immunity (57, 58). Numerous clinical and epidemiological investigations have consistently shown elevated levels of plasma inflammatory markers (59), such as C-reactive protein (60), high-sensitivity C-reactive protein (61), and interleukin-6 (62) in DKD patients. Although large-scale clinical trials specifically targeting therapies for DKD are lacking, the Finerenone in Reducing Kidney Failure and Disease Progression in Diabetic Kidney Disease (FIDELIO-DKD) trial has indicated that DKD patients derive greater benefit from anti-inflammatory treatment with Finerenone, a first novel, highly potent, selective mineralocorticoid receptor antagonist (63). This previous evidence suggests a potential synergistic relationship between inflammation levels and progression of DKD.

The SII is derived from the counts of three circulating immune cell types: neutrophils, lymphocytes, and platelets. It provides a comprehensive assessment of immune and inflammatory status, offering more clinical insights than single or dual peripheral blood parameters. Elevated SII levels often coincide with thrombocytosis, neutrophilia, or lymphopenia, reflecting heightened inflammatory responses and serving as a valuable diagnostic biomarker for systemic inflammatory activity (44). Particularly, numerous studies have underscored SII’s predictive capacity, linking higher SII levels to increased risks of various renal disease subtypes, including contrast-induced nephropathy (64), renal cell carcinoma (65), and peritoneal dialysis-treated chronic kidney disease patients (66). Moreover, several previous studies have shown the close relationship between SII and the incidence and severity of DKD, suggesting SII as a widely available, non-invasive, cost-effective, and straightforward approach to detecting and monitoring DKD (54, 55, 67). Accordingly, the therapeutic potential of anti-inflammation-based regimen is promising, emphasizing the importance of addressing systemic inflammation for better mortality risk prevention and prediction of DKD. Nevertheless, the relation between SII and clinical outcomes in DKD individuals remains largely undetermined.

In our current study, we for the first time identified a positive association between SII and increased risk of all-cause mortality, coupled with a linear association between SII and increased risk of cardio-cerebrovascular disease mortality, which underscores the value of SII in identifying high-risk individuals in DKD populations, thereby enabling early intervention. It is noteworthy that we observed a U-shaped relation between SII and all-cause mortality. Specifically, below the threshold value of 5.82 for lnSII, higher SII was significantly associated with lower all-cause mortality, while above the thresholds, SII was positively associated with all-cause mortality. Consistent with our present results, a previous study by Chen et al. revealed a U-shaped correlation between SII and all-cause mortality in populations with DM (43). Besides, Yan et al. observed that higher SII was closely associated with an increased risk of the presence and severity of DKD in Chinese population (67). These pieces of evidence, in conjunction with our findings, support the notion that SII holds promise as a potential biomarker for DKD. Importantly, both low and high SII levels might elevate mortality risk in individuals with DKD or DM, which aligns with the understanding that low platelet levels are typically associated with a heightened risk of bleeding that could contribute to higher all-cause mortality. While our study contributes to understanding the relationship between SII and mortality, the precise mechanisms underlying this association remain enigmatic and warrant further investigation.

Our study revealed an intriguing interaction between the SII and BMI in our subgroup analysis, highlighting the intricate relationship between inflammation and obesity in the development of DKD (35). This finding supports previous research indicating a complex interplay between SII and BMI, with BMI serving as a critical mediator in the association between SII and the risk of DM (68). Additionally, Kong et al.’s study identified SII as an independent risk factor for both all-cause and CVD-specific mortality in obese populations (53). Furthermore, numerous preclinical studies have underscored the pivotal role of dysregulated inflammatory responses in obesity-related pathogenesis (69). Intriguingly, the paradoxical association was observed when we seek to investigate the independent impact of BMI on the prognosis among the DKD cohorts. As illustrated in **Supplementary Figure 1A**, the association between BMI and all-cause mortality in DKD cohorts exhibits a U-shaped pattern. Specifically, the relationship between BMI levels on a continuous scale and the risk of all-cause mortality in the DKD cohort is U-shaped (*P* for nonlinearity = 0.003); both low and high BMI levels were linked to an increased risk of all-cause mortality (**Supplementary Figure 1A**). The BMI level associated with the lowest risk of incident all-cause mortality was 35.04 in the fully adjusted analyses. In contrast, as shown in **Supplementary Figure 1B**, BMI appears to have no correlation with cardio-cerebrovascular mortality. The obesity paradox observed in our study among DKD subjects aligns with numerous previous studies that demonstrate a significant association between BMI and all-cause mortality risk in diabetes cohorts (70, 71), chronic kidney disease cohorts (72–74), and the general population (75). However, elucidating the precise mechanisms underlying this interplay between inflammation and obesity in exacerbating DKD pathogenesis warrants further investigation.

Our study presents several strengths. Firstly, we analyzed a substantial sample size of 3195 individuals, ensuring robust representation of the population. Secondly, meticulous attention was paid to controlling for confounding variables, enhancing the reliability of our findings. Thirdly, our investigation is pioneering in its exploration of both linear and nonlinear relationships between the SII and mortality in DKD populations, evolving methodologically over time. Lastly, SII serves as an easily accessible, cost-effective measure with potential therapeutic implications or as an early warning indicator.

However, several limitations warrant consideration. Firstly, the observational nature of our study precludes establishing causal relationships. Secondly, despite efforts to adjust for various confounding factors, the influence of unmeasured variables such as diabetes duration, dietary habits, and treatment modalities remains a concern. Thirdly, while SII offers ease of measurement, factors affecting neutrophil, lymphocyte, and platelet counts could introduce selection bias. Lastly, our reliance on data from a single blood test may not fully capture temporal fluctuations in SII levels due to the short lifespan of blood cells. Continuous monitoring could provide more robust evidence than a one-time assessment.

## 5 Conclusions

In conclusion, our study revealed a U-shaped relationship between the SII and all-cause mortality, with threshold values of 5.82 for lnSII. Additionally, higher concentrations of SII exhibited a linear association with increased risk of cardio-cerebrovascular disease mortality. These findings underscore the independent prognostic significance of SII for patients with DKD. However, further extensive prospective investigations are warranted to validate and consolidate our findings.

## Data Availability

The original contributions presented in the study are included in the article/**Supplementary Material**. Further inquiries can be directed to the corresponding authors.

## References

[1] Tuttle KR Agarwal R Alpers CE Bakris GL Brosius FC Kolkhof P. Molecular mechanisms and therapeutic targets for diabetic kidney disease. Kidney Int. (2022) 102:248–60. doi: 10.1016/j.kint.2022.05.012 35661785

[2] Chiu YW Teitelbaum I Misra M de Leon EM Adzize T Mehrotra R. Pill burden, adherence, hyperphosphatemia, and quality of life in maintenance dialysis patients. Clin J Am Soc Nephrol. (2009) 4:1089–96. doi: 10.2215/CJN.00290109 PMC2689877 19423571

[3] Yong DS Kwok AO Wong DM Suen MH Chen WT Tse DM. Symptom burden and quality of life in end-stage renal disease: a study of 179 patients on dialysis and palliative care. Palliat Med. (2009) 23:111–9. doi: 10.1177/0269216308101099 19153131

[4] Apostolou T Hutchison AJ Boulton AJ Chak W Vileikyte L Uttley L. Quality of life in CAPD, transplant, and chronic renal failure patients with diabetes. Ren Fail. (2007) 29:189–97. doi: 10.1080/08860220601098862 17365935

[5] McFarlane PA Tobe SW Culleton B. Improving outcomes in diabetes and chronic kidney disease: the basis for Canadian guidelines. Can J Cardiol. (2007) 23:585–90. doi: 10.1016/s0828-282x(07)70806-1 PMC2650765 17534468

[6] Cha J Han D. Health-related quality of life based on comorbidities among patients with end-stage renal disease. Osong Public Health Res Perspect. (2020) 11:194–200. doi: 10.24171/j.phrp.2020.11.4.08 32864310 PMC7442444

[7] Svensson M Eriksson JW. Insulin resistance in diabetic nephropathy–cause or consequence? Diabetes Metab Res Rev. (2006) 22:401–10. doi: 10.1002/dmrr.648 16703644

[8] Karalliedde J Gnudi L. Diabetes mellitus, a complex and heterogeneous disease, and the role of insulin resistance as a determinant of diabetic kidney disease. Nephrol Dial Transplant. (2016) 31:206–13. doi: 10.1093/ndt/gfu405 25550448

[9] Parvanova AI Trevisan R Iliev IP Dimitrov BD Vedovato M Tiengo A. Insulin resistance and microalbuminuria: a cross-sectional, case-control study of 158 patients with type 2 diabetes and different degrees of urinary albumin excretion. Diabetes. (2006) 55:1456–62. doi: 10.2337/db05-1484 16644705

[10] de Luca C Olefsky JM. Inflammation and insulin resistance. FEBS Lett. (2008) 582:97–105. doi: 10.1016/j.febslet.2007.11.057 18053812 PMC2246086

[11] Rehman K Akash MS. Mechanisms of inflammatory responses and development of insulin resistance: how are they interlinked? J BioMed Sci. (2016) 23:87. doi: 10.1186/s12929-016-0303-y 27912756 PMC5135788

[12] Navarro JF Mora C. Role of inflammation in diabetic complications. Nephrol Dial Transplant. (2005) 20:2601–4. doi: 10.1093/ndt/gfi155 16188894

[13] Shahzad K Fatima S Khawaja H Elwakiel A Gadi I Ambreen S. Podocyte-specific Nlrp3 inflammasome activation promotes diabetic kidney disease. Kidney Int. (2022) 102:766–79. doi: 10.1016/j.kint.2022.06.010 35779608

[14] Mezzano S Aros C Droguett A Burgos ME Ardiles L Flores C. NF-kappaB activation and overexpression of regulated genes in human diabetic nephropathy. Nephrol Dial Transplant. (2004) 19:2505–12. doi: 10.1093/ndt/gfh207 15280531

[15] Lin M Yiu WH Wu HJ Chan LY Leung JC Au WS. Toll-like receptor 4 promotes tubular inflammation in diabetic nephropathy. J Am Soc Nephrol. (2012) 23:86–102. doi: 10.1681/ASN.2010111210 22021706 PMC3269929

[16] Han YC Tang SQ Liu YT Li AM Zhan M Yang M. AMPK agonist alleviate renal tubulointerstitial fibrosis via activating mitophagy in high fat and streptozotocin induced diabetic mice. Cell Death Dis. (2021) 12:925. doi: 10.1038/s41419-021-04184-8 34628484 PMC8502176

[17] Hasegawa S Tanaka T Saito T Fukui K Wakashima T Susaki EA. The oral hypoxia-inducible factor prolyl hydroxylase inhibitor enarodustat counteracts alterations in renal energy metabolism in the early stages of diabetic kidney disease. Kidney Int. (2020) 97:934–50. doi: 10.1016/j.kint.2019.12.007 32171449

[18] Santilli F Simeone P Liani R Davi G. Platelets and diabetes mellitus. Prostaglandins Other Lipid Mediat. (2015) 120:28–39. doi: 10.1016/j.prostaglandins.2015.05.002 25986598

[19] Pretorius E. Platelets as potent signaling entities in type 2 diabetes mellitus. Trends Endocrinol Metab. (2019) 30:532–45. doi: 10.1016/j.tem.2019.05.003 31196615

[20] Zhang H Chen H Wu X Sun T Fan M Tong H. Tetramethylpyrazine alleviates diabetes-induced high platelet response and endothelial adhesion via inhibiting NLRP3 inflammasome activation. Phytomedicine. (2022) 96:153860. doi: 10.1016/j.phymed.2021.153860 34836743

[21] Rustiasari UJ Roelofs JJ. The role of platelets in diabetic kidney disease. Int J Mol Sci. (2022) 23. doi: 10.3390/ijms23158270 PMC9368651 35955405

[22] Hafez HM Abdel-Hakeem EA Hassanein H. Rupatadine, a dual antagonist of histamine and platelet-activating factor (PAF), attenuates experimentally induced diabetic nephropathy in rats. Naunyn Schmiedebergs Arch Pharmacol. (2020) 393:1487–500. doi: 10.1007/s00210-020-01856-8 32200462

[23] Gruden G Cavallo-Perin P Romagnoli R Ruiu G Pagano G. Plasma beta-thromboglobulin and platelet factor 4 are not increased in insulin-dependent diabetic patients with microalbuminuria. Acta Diabetol. (1994) 31:130–2. doi: 10.1007/BF00570365 7827349

[24] Chen J Tan W. Platelet activation and immune response in diabetic microangiopathy. Clin Chim Acta. (2020) 507:242–7. doi: 10.1016/j.cca.2020.04.042 32376322

[25] Chaudhary PK Kim S Kim S. An insight into recent advances on platelet function in health and disease. Int J Mol Sci. (2022) 23. doi: 10.3390/ijms23116022 PMC9181192 35682700

[26] Chen J Liu Q He J Li Y. Immune responses in diabetic nephropathy: Pathogenic mechanisms and therapeutic target. Front Immunol. (2022) 13:958790. doi: 10.3389/fimmu.2022.958790 36045667 PMC9420855

[27] Wu CC Sytwu HK Lin YF. Cytokines in diabetic nephropathy. Adv Clin Chem. (2012) 56:55–74. doi: 10.1016/b978-0-12-394317-0.00014-5 22397028

[28] Manne BK Xiang SC Rondina MT. Platelet secretion in inflammatory and infectious diseases. Platelets. (2017) 28:155–64. doi: 10.1080/09537104.2016.1240766 PMC5734920 27848259

[29] Zhang R Chen J Xiong Y Wang L Huang X Sun T. Increased neutrophil count Is associated with the development of chronic kidney disease in patients with diabetes. J Diabetes. (2022) 14:442–54. doi: 10.1111/1753-0407.13292 PMC9310049 35789114

[30] Huang W Huang J Liu Q Lin F He Z Zeng Z. Neutrophil-lymphocyte ratio is a reliable predictive marker for early-stage diabetic nephropathy. Clin Endocrinol (Oxf). (2015) 82:229–33. doi: 10.1111/cen.12576 25088518

[31] Liu J Liu X Li Y Quan J Wei S An S. The association of neutrophil to lymphocyte ratio, mean platelet volume, and platelet distribution width with diabetic retinopathy and nephropathy: a meta-analysis. Biosci Rep. (2018) 38. doi: 10.1042/BSR20180172 PMC6019380 29581246

[32] Chung FM Tsai JC Chang DM Shin SJ Lee YJ. Peripheral total and differential leukocyte count in diabetic nephropathy: the relationship of plasma leptin to leukocytosis. Diabetes Care. (2005) 28:1710–7. doi: 10.2337/diacare.28.7.1710 15983324

[33] Schmidt MI Duncan BB Sharrett AR Lindberg G Savage PJ Offenbacher S. Markers of inflammation and prediction of diabetes mellitus in adults (Atherosclerosis Risk in Communities study): a cohort study. Lancet. (1999) 353:1649–52. doi: 10.1016/s0140-6736(99)01046-6 10335783

[34] Hu B Yang XR Xu Y Sun YF Sun C Guo W. Systemic immune-inflammation index predicts prognosis of patients after curative resection for hepatocellular carcinoma. Clin Cancer Res. (2014) 20:6212–22. doi: 10.1158/1078-0432.CCR-14-0442 25271081

[35] Zhao Y Shao W Zhu Q Zhang R Sun T Wang B. Association between systemic immune-inflammation index and metabolic syndrome and its components: results from the National Health and Nutrition Examination Survey 2011-2016. J Transl Med. (2023) 21:691. doi: 10.1186/s12967-023-04491-y 37794370 PMC10548719

[36] Ye Z Hu T Wang J Xiao R Liao X Liu M. Systemic immune-inflammation index as a potential biomarker of cardiovascular diseases: A systematic review and meta-analysis. Front Cardiovasc Med. (2022) 9:933913. doi: 10.3389/fcvm.2022.933913 36003917 PMC9393310

[37] Zhao E Cheng Y Yu C Li H Fan X. The systemic immune-inflammation index was non-linear associated with all-cause mortality in individuals with nonalcoholic fatty liver disease. Ann Med. (2023) 55:2197652. doi: 10.1080/07853890.2023.2197652 37052341 PMC10115001

[38] Nie Y Zhou H Wang J Kan H. Association between systemic immune-inflammation index and diabetes: a population-based study from the NHANES. Front Endocrinol (Lausanne). (2023) 14:1245199. doi: 10.3389/fendo.2023.1245199 38027115 PMC10644783

[39] Qin Z Li H Wang L Geng J Yang Q Su B. Systemic immune-inflammation index is associated with increased urinary albumin excretion: A population-based study. Front Immunol. (2022) 13:863640. doi: 10.3389/fimmu.2022.863640 35386695 PMC8977553

[40] Wang S Pan X Jia B Chen S. Exploring the correlation between the systemic immune inflammation index (SII), systemic inflammatory response index (SIRI), and type 2 diabetic retinopathy. Diabetes Metab Syndr Obes. (2023) 16:3827–36. doi: 10.2147/DMSO.S437580 PMC10683512 38033457

[41] Li J Zhang X Zhang Y Dan X Wu X Yang Y. Increased systemic immune-inflammation index was associated with type 2 diabetic peripheral neuropathy: A cross-sectional study in the Chinese population. J Inflamm Res. (2023) 16:6039–53. doi: 10.2147/JIR.S433843 PMC10723178 38107379

[42] Ma LL Xiao HB Zhang J Liu YH Hu LK Chen N. Association between systemic immune inflammatory/inflammatory response index and hypertension: A cohort study of functional community. Nutr Metab Cardiovasc Dis. (2024) 34:334–42. doi: 10.1016/j.numecd.2023.09.025 38000992

[43] Chen C Chen Y Gao Q Wei Q. Association of systemic immune inflammatory index with all-cause and cause-specific mortality among individuals with type 2 diabetes. BMC Cardiovasc Disord. (2023) 23:596. doi: 10.1186/s12872-023-03638-5 38057733 PMC10702126

[44] Wang H Nie H Bu G Tong X Bai X. Systemic immune-inflammation index (SII) and the risk of all-cause, cardiovascular, and cardio-cerebrovascular mortality in the general population. Eur J Med Res. (2023) 28:575. doi: 10.1186/s40001-023-01529-1 38066657 PMC10709886

[45] Johnson CL Paulose-Ram R Ogden CL Carroll MD Kruszon-Moran D Dohrmann SM. National health and nutrition examination survey: analytic guidelines, 1999-2010. Vital Health Stat. (2013) 2:1–24. 25090154

[46] American Diabetes Association Professional Practice C. 2. Diagnosis and classification of diabetes: standards of care in diabetes-2024. Diabetes Care. (2024) 47:S20–42. doi: 10.2337/dc24-S002 PMC10725812 38078589

[47] Chu CD Xia F Du Y Singh R Tuot DS Lamprea-Montealegre JA. Estimated prevalence and testing for albuminuria in US adults at risk for chronic kidney disease. JAMA Netw Open. (2023) 6:e2326230. doi: 10.1001/jamanetworkopen.2023.26230 37498594 PMC10375308

[48] Kidney Disease G. Improving global outcomes diabetes work: KDIGO 2022 clinical practice guideline for diabetes management in chronic kidney disease. Kidney Int. (2022) 102:S1–S127. doi: 10.1016/j.kint.2022.06.008 36272764

[49] Collaborators GBDO Afshin A Forouzanfar MH. Health Effects of Overweight and Obesity in 195 Countries over 25 Years. N Engl J Med. (2017) 377:13–27. 28604169 10.1056/NEJMoa1614362 PMC5477817

[50] Hakeem FF Bernabe E Sabbah W. Association between oral health and frailty among American older adults. J Am Med Dir Assoc. (2021) 22:559–563.e2. doi: 10.1016/j.jamda.2020.07.023 32859517

[51] Unger T Borghi C Charchar F Khan NA Poulter NR Prabhakaran D. 2020 International society of hypertension global hypertension practice guidelines. Hypertension. (2020) 75:1334–57. doi: 10.1161/HYPERTENSIONAHA.120.15026 32370572

[52] Yang C Yang Q Xie Z Peng X Liu H Xie C. Association of systemic immune-inflammation-index with all-cause and cause-specific mortality among type 2 diabetes: a cohort study base on population. Endocrine. (2024) 84:399–411. doi: 10.1007/s12020-023-03587-1 38048013 PMC11076376

[53] Kong F Huang J Xu C Huang T Wen G Cheng W. System inflammation response index: a novel inflammatory indicator to predict all-cause and cardiovascular disease mortality in the obese population. Diabetol Metab Syndr. (2023) 15:195. doi: 10.1186/s13098-023-01178-8 37821960 PMC10566161

[54] Liu W Zheng S Du X. Association of systemic immune-inflammation index and systemic inflammation response index with diabetic kidney disease in patients with type 2 diabetes mellitus. Diabetes Metab Syndr Obes. (2024) 17:517–31. doi: 10.2147/DMSO.S447026 PMC10849098 38327734

[55] Guo W Song Y Sun Y Du H Cai Y You Q. Systemic immune-inflammation index is associated with diabetic kidney disease in Type 2 diabetes mellitus patients: Evidence from NHANES 2011-2018. Front Endocrinol (Lausanne). (2022) 13:1071465. doi: 10.3389/fendo.2022.1071465 36561561 PMC9763451

[56] Fu J Sun Z Wang X Zhang T Yuan W Salem F. The single-cell landscape of kidney immune cells reveals transcriptional heterogeneity in early diabetic kidney disease. Kidney Int. (2022) 102:1291–304. doi: 10.1016/j.kint.2022.08.026 PMC9691617 36108806

[57] Wilson PC Muto Y Wu H Karihaloo A Waikar SS Humphreys BD. Multimodal single cell sequencing implicates chromatin accessibility and genetic background in diabetic kidney disease progression. Nat Commun. (2022) 13:5253. doi: 10.1038/s41467-022-32972-z 36068241 PMC9448792

[58] Wu H Gonzalez Villalobos R Yao X Reilly D Chen T Rankin M. Mapping the single-cell transcriptomic response of murine diabetic kidney disease to therapies. Cell Metab. (2022) 34:1064–1078.e6. doi: 10.1016/j.cmet.2022.05.010 35709763 PMC9262852

[59] Rayego-Mateos S Rodrigues-Diez RR Fernandez-Fernandez B Mora-Fernandez C Marchant V Donate-Correa J. Targeting inflammation to treat diabetic kidney disease: the road to 2030. Kidney Int. (2023) 103:282–96. doi: 10.1016/j.kint.2022.10.030 36470394

[60] Festa A D'Agostino R Howard G Mykkanen L Tracy RP Haffner SM. Inflammation and microalbuminuria in nondiabetic and type 2 diabetic subjects: The Insulin Resistance Atherosclerosis Study. Kidney Int. (2000) 58:1703–10. doi: 10.1046/j.1523-1755.2000.00331.x 11012904

[61] Sinha SK Nicholas SB Sung JH Correa A Rajavashisth TB Norris KC. hs-CRP is associated with incident diabetic nephropathy: findings from the jackson heart study. Diabetes Care. (2019) 42:2083–9. doi: 10.2337/dc18-2563 PMC6804609 31511234

[62] Navarro JF Mora C Gomez M Muros M Lopez-Aguilar C Garcia J. Influence of renal involvement on peripheral blood mononuclear cell expression behaviour of tumour necrosis factor-alpha and interleukin-6 in type 2 diabetic patients. Nephrol Dial Transplant. (2008) 23:919–26. doi: 10.1093/ndt/gfm674 17911088

[63] Bakris GL Agarwal R Anker SD Pitt B Ruilope LM Rossing P. Effect of finerenone on chronic kidney disease outcomes in type 2 diabetes. N Engl J Med. (2020) 383:2219–29. doi: 10.1056/NEJMoa2025845 33264825

[64] Kelesoglu S Yilmaz Y Elcik D Cetinkaya Z Inanc MT Dogan A. Systemic immune inflammation index: A novel predictor of contrast-induced nephropathy in patients with non-ST segment elevation myocardial infarction. Angiology. (2021) 72:889–95. doi: 10.1177/00033197211007738 33827291

[65] Jin M Yuan S Yuan Y Yi L. Prognostic and clinicopathological significance of the systemic immune-inflammation index in patients with renal cell carcinoma: A meta-analysis. Front Oncol. (2021) 11:735803. doi: 10.3389/fonc.2021.735803 34950577 PMC8689141

[66] Tang R Chen J Zhou Q Deng J Zhan X Wang X. Association between systemic immune inflammation Index and all-cause mortality in incident peritoneal dialysis-treated CKD patients: a multi-center retrospective cohort study. BMC Nephrol. (2024) 25:8. doi: 10.1186/s12882-023-03451-4 38172773 PMC10765751

[67] Yan P Yang Y Zhang X Zhang Y Li J Wu Z. Association of systemic immune-inflammation index with diabetic kidney disease in patients with type 2 diabetes: a cross-sectional study in Chinese population. Front Endocrinol (Lausanne). (2023) 14:1307692. doi: 10.3389/fendo.2023.1307692 38239983 PMC10795757

[68] Chen Y Huang R Mai Z Chen H Zhang J Zhao L. Association between systemic immune-inflammatory index and diabetes mellitus: mediation analysis involving obesity indicators in the NHANES. Front Public Health. (2023) 11:1331159. doi: 10.3389/fpubh.2023.1331159 38269383 PMC10806151

[69] Rocha VZ Folco EJ. Inflammatory concepts of obesity. Int J Inflam. (2011) 2011:529061. doi: 10.4061/2011/529061 21837268 PMC3151511

[70] Zhao W Katzmarzyk PT Horswell R Wang Y Li W Johnson J. Body mass index and the risk of all-cause mortality among patients with type 2 diabetes mellitus. Circulation. (2014) 130:2143–51. doi: 10.1161/CIRCULATIONAHA.114.009098 PMC4302029 25378546

[71] Jackson CL Yeh HC Szklo M Hu FB Wang NY Dray-Spira R. Body-mass index and all-cause mortality in US adults with and without diabetes. J Gen Intern Med. (2014) 29:25–33. doi: 10.1007/s11606-013-2553-7 23929218 PMC3889975

[72] Navaneethan SD Schold JD Arrigain S Kirwan JP Nally JV Jr. Body mass index and causes of death in chronic kidney disease. Kidney Int. (2016) 89:675–82. doi: 10.1016/j.kint.2015.12.002 PMC4757850 26880461

[73] Ahmadi SF Zahmatkesh G Ahmadi E Streja E Rhee CM Gillen DL. Association of body mass index with clinical outcomes in non-dialysis-dependent chronic kidney disease: A systematic review and meta-analysis. Cardiorenal Med. (2015) 6:37–49. doi: 10.1159/000437277 27194995 PMC4698625

[74] Kovesdy CP Anderson JE Kalantar-Zadeh K. Paradoxical association between body mass index and mortality in men with CKD not yet on dialysis. Am J Kidney Dis. (2007) 49:581–91. doi: 10.1053/j.ajkd.2007.02.277 17472839

[75] Kim NH Lee J Kim TJ Kim NH Choi KM Baik SH. Body mass index and mortality in the general population and in subjects with chronic disease in Korea: A nationwide cohort study (2002-2010). PloS One. (2015) 10:e0139924. doi: 10.1371/journal.pone.0139924 26462235 PMC4604086

