# Supplementary figures and images for: Association of systemic immune-inflammation index with all-cause and cardio-cerebrovascular mortality in individuals with diabetic kidney disease: evidence from NHANES 1999-2018
Source: Front Endocrinol (Lausanne). 2024 Nov 26;15:1399832. doi: 10.3389/fendo.2024.1399832 (PMC11628304; doi:10.3389/fendo.2024.1399832)

Supplementary Figure 1

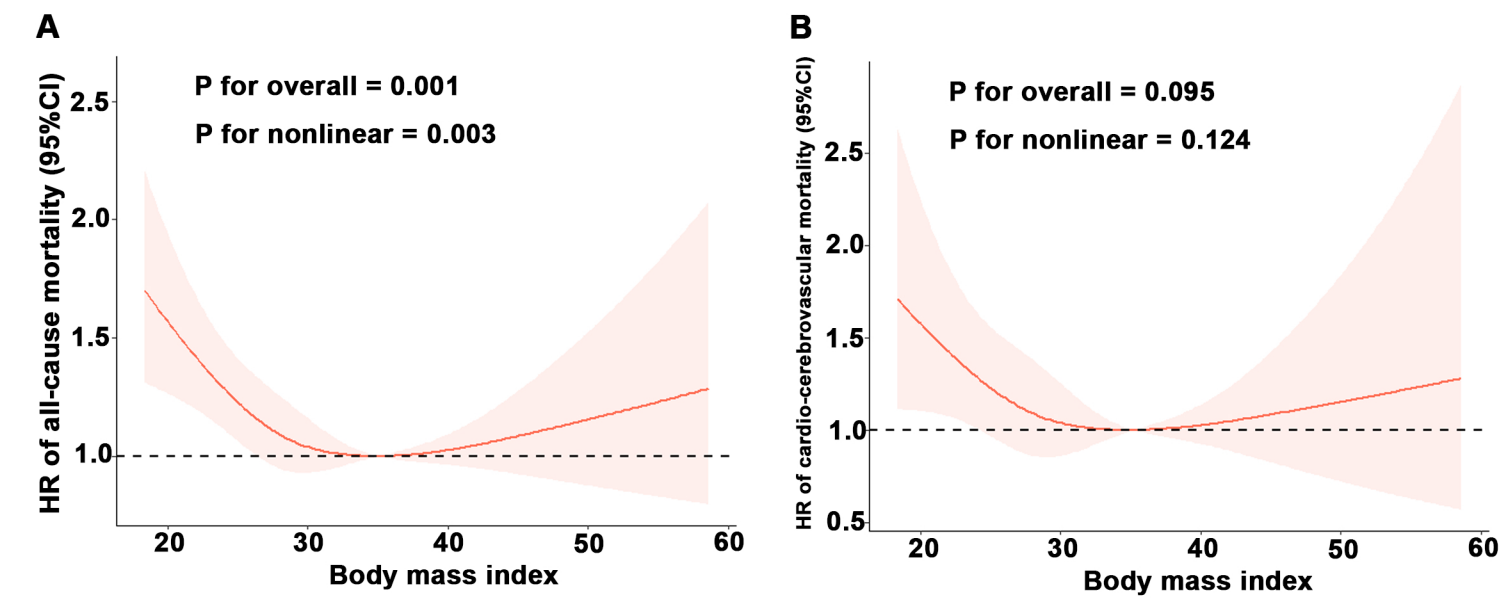

Supplement: Supplementary file 1 [file Image1.pdf]
